# Reproductive factors and risk of mortality in the European Prospective Investigation into Cancer and Nutrition; a cohort study

**DOI:** 10.1186/s12916-015-0484-3

**Published:** 2015-10-30

**Authors:** Melissa A. Merritt, Elio Riboli, Neil Murphy, Mai Kadi, Anne Tjønneland, Anja Olsen, Kim Overvad, Laure Dossus, Laureen Dartois, Françoise Clavel-Chapelon, Renée T. Fortner, Verena A. Katzke, Heiner Boeing, Antonia Trichopoulou, Pagona Lagiou, Dimitrios Trichopoulos, Domenico Palli, Sabina Sieri, Rosario Tumino, Carlotta Sacerdote, Salvatore Panico, H. Bas Bueno-de-Mesquita, Petra H. Peeters, Eiliv Lund, Aurelie Nakamura, Elisabete Weiderpass, J. Ramón Quirós, Antonio Agudo, Esther Molina-Montes, Nerea Larrañaga, Miren Dorronsoro, Lluís Cirera, Aurelio Barricarte, Åsa Olsson, Salma Butt, Annika Idahl, Eva Lundin, Nicholas J. Wareham, Timothy J. Key, Paul Brennan, Pietro Ferrari, Petra A. Wark, Teresa Norat, Amanda J. Cross, Marc J. Gunter

**Affiliations:** Department of Epidemiology and Biostatistics, School of Public Health, Imperial College London, Norfolk Place, London, W2 1PG UK; Danish Cancer Society Research Center, Strandboulevarden 49, DK-2100 Copenhagen, Denmark; Department of Public Health, Section for Epidemiology, Aarhus University, Bartholins Allé 2 – Building 1260, DK-8000 Aarhus, Denmark; Inserm, Centre for research in Epidemiology and Population Health (CESP), U1018, Nutrition, Hormones and Women’s Health team, F-94805 Villejuif, France; Université Paris Sud, UMRS 1018, F-94805 Villejuif, France; Institut Gustave Roussy, F-94805 Villejuif, France; Division of Cancer Epidemiology, German Cancer Research Center (DKFZ), Im Neuenheimer Feld 280, 69120 Heidelberg, Germany; Department of Epidemiology, German Institute of Human Nutrition Potsdam-Rehbruecke, Arthur-Scheunert-Allee 114-116, 14558 Nuthetal, Germany; Hellenic Health Foundation, 13 Kaisareias Street, Athens, GR-115 27 Greece; Department of Hygiene, Epidemiology and Medical Statistics, University of Athens Medical School, 75 M Asias Street, Goudi GR-115 27, Athens, Greece; Bureau of Epidemiologic Research, Academy of Athens, 23 Alexandroupoleos Street, Athens, GR-115 27 Greece; Department of Epidemiology, Harvard School of Public Health, 677 Huntington Avenue, Boston, MA 02115 USA; Molecular and Nutritional Epidemiology Unit, Cancer Research and Prevention Institute – ISPO, Ponte Nuovo Palazzina 28 A “Mario Fiori”, Via delle Oblate 4, 50141 Florence, Italy; Epidemiology and Prevention Unit, Fondazione IRCCS Istituto Nazionale dei Tumori, Via Venezian, 1, 20133 Milan, Italy; Cancer Registry and Histopathology Unit, “Civic – M.P.Arezzo” Hospital, ASP, Via Dante N° 109, 97100 Ragusa, Italy; Unit of Cancer Epidemiology, Citta’ della Salute e della Scienza Hospital- University of Turin and Center for Cancer Prevention (CPO), Via Santena 7, 10126 Turin, Italy; Dipartimento di Medicina Clinica e Chirurgia, Federico II University, via Pansini 5, 80131 Naples, Italy; Department for Determinants of Chronic Diseases (DCD), National Institute for Public Health and the Environment (RIVM), PO Box 1, 3720 BA Bilthoven, The Netherlands; Department of Gastroenterology and Hepatology, University Medical Centre Utrecht, Heidelberglann 100, 3584 CX Utrecht, The Netherlands; Department of Social and Preventive Medicine, Faculty of Medicine, University of Malaya, 50603 Kuala Lumpur, Malaysia; Department of Epidemiology, Julius Center for Health Sciences and Primary Care, University Medical Center, Huispost Str. 6.131, PO Box 85500, 3508 GA Utrecht, The Netherlands; Department of Community Medicine, Faculty of Health Sciences, University of Tromsø, The Arctic University of Norway, Breivika, N-9037 Tromsø Norway; Department of Research, Cancer Registry of Norway, 0310 Oslo, Norway; Department of Medical Epidemiology and Biostatistics, Karolinska Institutet, Stockholm, 17177 Sweden; Genetic Epidemiology Group, Folkhälsan Research Center, Helsinki, FI-00290 Finland; Public Health Directorate, Asturias, Ciriaco Miguel Vigil St, 9, 33006 Oviedo, Spain; Unit of Nutrition and Cancer, IDIBELL, Catalan Institute of Oncology-ICO, L’Hospitalet de Llobregat, Barcelona, 08908 Spain; Escuela Andaluza de Salud Pública, Instituto de Investigación Biosanitaria ibs and Hospitales Universitarios de Granada/Universidad de Granada, Cuesta del Observatorio, 4, Campus Universitario de Cartuja, 18080 Granada, Spain; CIBER de Epidemiología y Salud Pública (CIBERESP), Melchor Fernández Almagro, 3-5, 28029 Madrid, Spain; Public Health Division of Gipuzkoa, BIODonostia Research Institute, Basque Health Department, Avenida de Navarra, 4-20013 Donostia San Sebastian, Spain; Public Health Direction and Biodonostia-Ciberesp, Basque Regional Health Department, 01010 Vitoria, Spain; Department of Epidemiology, Murcia Regional Health Council, IMIB - Arrixaca, Ronda de Levante 11, 30008 Murcia, Spain; Navarre Public Health Institute, Leyre 15, 31003 Pamplona, Spain; Institute of Clinical Sciences, Malmö and Department of Surgery, Lund University, Skane University Hospital, SE-205 02 Malmö, Sweden; Department of Clinical Sciences, Obstetrics and Gynecology, Umeå University, SE-901 87 Umeå, Sweden; Department of Public Health and Clinical Medicine, Nutritional Research, Umeå University, SE-901 87 Umeå, Sweden; Department of Medical Biosciences, Pathology, Umeå University, SE-901 87 Umeå, Sweden; MRC Epidemiology Unit, University of Cambridge, Institute of Metabolic Science, Addenbrooke’s Hospital, Hills Road, PO Box 285, Cambridge, CB2 0QQ UK; Cancer Epidemiology Unit, Nuffield Department of Population Health, Richard Doll Building, University of Oxford, Oxford, OX3 7LF UK; International Agency for Research on Cancer, 150 Cours Albert-Thomas, 69372 Lyon, Cedex 08 France

**Keywords:** Age at menarche, Age at menopause, Breastfeeding, Mortality, Oral contraceptives, Parity

## Abstract

**Background:**

Reproductive events are associated with important physiologic changes, yet little is known about how reproductive factors influence long-term health in women. Our objective was to assess the relation of reproductive characteristics with all-cause and cause-specific mortality risk.

**Methods:**

The analysis was performed within the European Investigation into Cancer and Nutrition prospective cohort study, which enrolled >500,000 women and men from 1992 to 2000, who were residing in a given town/geographic area in 10 European countries. The current analysis included 322,972 eligible women aged 25–70 years with 99 % complete follow-up for vital status. We assessed reproductive characteristics reported at the study baseline including parity, age at the first birth, breastfeeding, infertility, oral contraceptive use, age at menarche and menopause, total ovulatory years, and history of oophorectomy/hysterectomy. Hazard ratios (HRs) and 95 % confidence intervals (CIs) for mortality were determined using Cox proportional hazards regression models adjusted for menopausal status, body mass index, physical activity, education level, and smoking status/intensity and duration.

**Results:**

During a mean follow-up of 12.9 years, 14,383 deaths occurred. The HR (95 % CI) for risk of all-cause mortality was lower in parous versus nulliparous women (0.80; 0.76–0.84), in women who had ever versus never breastfed (0.92; 0.87–0.97), in ever versus never users of oral contraceptives (among non-smokers; 0.90; 0.86–0.95), and in women reporting a later age at menarche (≥15 years versus <12; 0.90; 0.85–0.96; *P* for trend = 0.038).

**Conclusions:**

Childbirth, breastfeeding, oral contraceptive use, and a later age at menarche were associated with better health outcomes. These findings may contribute to the development of improved strategies to promote better long-term health in women.

**Electronic supplementary material:**

The online version of this article (doi:10.1186/s12916-015-0484-3) contains supplementary material, which is available to authorized users.

## Background

Reproductive events represent significant biological milestones in a woman’s life and are associated with profound physiologic and endocrinologic changes. It is recognized that reproductive factors influence the risk of developing reproductive-related cancers; for example, age at menarche, use of oral contraceptives (OCs), parity, breastfeeding, and age at menopause are associated with risk of developing cancers of the breast, endometrium, and ovary [1–3]. Analyses of reproductive parameters in relation to all-cause and cause-specific mortality risk can provide further insights to understand how reproductive factors may influence the general long-term health of women.

Most [4–7], but not all [8], relevant studies reported that an earlier age at menarche was associated with a higher risk for all-cause mortality and/or selected cause-specific mortalities. Previous studies have reported no association [8–10], or an inverse association [11, 12], for ever versus never use of OCs with risk of all-cause mortality and/or mortality from cancer or cardiovascular disease. The association between parity and risk of all-cause and cause-specific mortality is uncertain due to inconsistent results across studies possibly because many [13–18], but not all [19–21], had incomplete information on other chronic disease risk factors which may confound the relationships, such as body mass index (BMI) and smoking habits. Although fewer studies have examined breastfeeding and mortality risk, recent analyses highlighted a lower risk of ischaemic heart disease [8] or circulatory/cardiovascular disease mortality [22, 23] among parous women who had ever versus never breastfed. Finally, some investigations reported that an early age at menopause was associated with a higher risk of mortality particularly from cardiovascular outcomes [24–27].

As reproductive factors are intricately linked, we evaluated several characteristics in relation to risk of all-cause and cause-specific mortality while adjusting for potential confounding factors, including BMI, physical activity, smoking, and education level. This study provides evidence from a large European prospective study on how common reproductive factors may influence the long-term health of women.

## Methods

### Study population

The European Prospective Investigation into Cancer and Nutrition (EPIC) study includes 518,408 participants (366,040 women) aged 25–70 years at enrolment (1992–2000). The cohort and data collection have been previously described [28, 29]. Briefly, study participants were recruited from the general population if they resided in a particular town or province in 23 centres in 10 European countries (Denmark, France, Germany, Greece, Italy, the Netherlands, Norway, Spain, Sweden, and the United Kingdom). Exceptions to this were the French cohort, which includes female members of the health insurance for teachers; components of the Italian (Ragusa and Turin) and Spanish cohorts that included members of local blood donor associations; Utrecht (the Netherlands) and Florence (Italy) cohorts, from where women attending population-based mammographic screening programs were recruited; Oxford (United Kingdom), where half of the cohort included “health conscious” participants from England, Wales, Scotland, and Northern Ireland who did not eat meat; and the cohorts of France, Norway, Utrecht (the Netherlands), and Naples (Italy) which included women only. Data on diet, lifestyle characteristics, and medical history were collected using baseline questionnaire and interview data. Informed consent was provided by all participants and ethical approval was obtained from the internal review board of the International Agency for Research on Cancer and from local ethics committees in the participating countries. The full list of all local ethics committees is provided in Additional file 1.

Exclusions at the study baseline were men; participants reporting prevalent diseases that could influence important confounders, diabetes (n = 8,298), myocardial infarction/heart attack (n = 2,063), angina (n = 3,275), stroke (n = 1,920), or cancer (except non-melanoma skin cancer; n = 18,649); participants who were missing a lifestyle questionnaire (n = 536), vital status (n = 743), or their date of death (n = 216); those who reported having never menstruated (n = 37) or were missing all of the following: age at menarche or menopause (postmenopausal women only), number of full term pregnancies (FTPs), age at first and last FTP, OC use, and duration of breastfeeding (n = 7,331). Finally, 322,972 women were included in the analysis.

### Exposure and covariate assessment

Reproductive characteristics assessed at the study baseline included parity (live/still births only), number of FTPs, age at first FTP, breastfeeding, infertility, OC ever use and current use, duration of OC use up until the time of recruitment, age at menarche, age at natural menopause, total ovulatory years, and history of oophorectomy or hysterectomy. Information on breastfeeding was only available for the first three and the last FTP, therefore the breastfeeding duration was calculated as the sum of these pregnancies and for women reporting >4 FTPs as the number of pregnancies multiplied by the mean duration of breastfeeding per child. Women with infertility were defined as those who had ever seen a doctor for fertility problems or if they reported a diagnosis, treatment, or surgery for fertility problems. OC formulations have changed over time; most notably, current OCs have lower estrogen doses and may contain as little as 20 μg ethinyl estradiol, while OCs that were prescribed before 1970 were typically high dose preparations (~100 μg ethinyl estradiol) [30]. Data on OC formulation were not available in the EPIC study; therefore, we carried out sensitivity analyses of OC use after stratifying by calendar year of first use, before 1970 (high dose), 1970–79 (medium dose), and 1980 and later (low dose). The age at natural menopause was defined as the age at the last menstrual period and participants who reported a surgical menopause (due to hysterectomy or oophorectomy) that occurred before reaching their age at natural menopause or participants missing the date of their surgical menopause were excluded from this analysis. Age at menopause was categorized consistently with previous EPIC study reports [31, 32] and the largest category (46–50 years) was set as the referent group. Ovulatory years were calculated as the difference between a participant’s age at menopause (postmenopausal) or their age at recruitment (premenopausal or perimenopausal/unknown menopause), and their age at menarche, minus the length of time that a woman was pregnant or using OCs. Anthropometric data [33], physical activity incorporating occupational and recreational activities [34], smoking status/intensity and duration, marital status, and education level at the study baseline also were assessed.

### Documentation of mortality endpoints

Follow-up of study subjects for vital status, cause, and date of death commenced in the mid-1990s and the current study uses the most recent data from the follow-up cycle completed in 2010. Vital status data were collected using record linkages with cancer registries, boards of health, and death indices in Denmark, Italy (except Naples), the Netherlands, Norway, Spain, Sweden, and the United Kingdom or through active follow-up (inquiries by mail or telephone to participants, municipal registries, regional health departments, physicians, and hospitals) in Germany, Greece, Naples, Italy, and France following standardized guidelines for the collection of endpoint data in the EPIC Study (IARC, 1998, unpublished). Procedures to ensure that valid and complete active follow-up data were collected were previously described for the German [35], Greek [36], Naples [37], and French [38] subcohorts. Causes of death were coded according to the 10^th^ revision of the International Statistical Classification of Diseases, Injuries, and Causes of Death. For cause-specific mortality analyses, deaths were grouped into categories representing the most common causes of death (cancer, circulatory disease), and categories were further subdivided into the most commonly occurring disease subgroups; breast cancer, cerebrovascular, and ischaemic heart disease.

### Statistical analyses

Cox proportional hazards regression modelling using age as the underlying time metric with the subjects’ age at recruitment as the entry time and their age at death or censoring, emigration, or last complete follow-up, whichever occurred first, as the exit time, were used to estimate hazard ratios (HRs) and 95 % confidence intervals (CIs) for the associations between reproductive characteristics and mortality risk. To account for differences across study centres in the timing to report causes of death, in cause-specific analyses the follow-up dates were truncated to when 80 % of the causes of death at each centre were known; specifically, June 2005 (Cambridge), December 2006 (France, Varese, Turin, Naples, Granada, Murcia, Malmo, Denmark), December 2007 (Florence, San Sebastian, Umeå, Norway), December 2008 (Ragusa, Asturias, Navarra, the Netherlands), and June 2009 (Oxford). For Germany and Greece, the end of the follow-up was the last known date of contact or death; this extended to July 2010 (Germany) and December 2009 for Greece.

Multivariate analyses were adjusted for important confounders that were selected *a priori*; menopausal status, BMI, physical activity, education level, and smoking status/intensity and duration while including an indicator category for missing data, and all models were stratified by the study centre and the participant’s age at recruitment. Sensitivity analyses that excluded individuals with missing values for these covariates showed similar results; these data are not presented here. We examined alcohol intake as a potential confounder by classifying individuals into quartiles based on their levels of ethanol intake in grams per day but did not include this in the final models because the HRs were not altered by >10 % [39]. Continuous variables were modelled to calculate a *P* for trend.

We carried out further separate analyses of all-cause mortality risk to examine the following associations: parity (parous versus nulliparous) when stratifying by marital status (never, ever married); number of FTPs when stratifying by BMI (<25 kg/m^2^, ≥25), since having more children may lead to weight gain; age at menarche after stratifying by BMI because an early age at menarche has been linked to a higher BMI in adulthood; breastfeeding duration after stratifying by the number of FTPs (1–2 FTPs, >2); and age at menopause and ever use of OCs were assessed separately after stratifying by smoking status. In each of these models, a *P* for statistical interaction was calculated using a likelihood ratio test to compare multivariate models with and without multiplicative interaction terms. Additional sensitivity analyses were conducted after restricting to women who were postmenopausal at recruitment, or without excluding participants who reported prevalent conditions. In analyses of age at menopause, we conducted further sensitivity analysis of breast cancer mortality without excluding women who had a surgically-induced menopause. The proportional hazards assumption was verified using the Grambsch and Therneau [40] method. A two tailed *P* <0.05 was considered statistically significant. Analyses were performed using the survival package [41] in R (version 3.0.2) [42].

## Results

### Baseline characteristics

After a mean follow-up of 12.9 (SD = 2.3) years, 14,383 all-cause deaths were identified, including 5,938 cancer deaths and 2,404 deaths from circulatory diseases. The distribution of most reproductive characteristics was similar across countries (Table 1) although there were differences in the proportion of women who used OCs (11–40 % in Greece, Spain, and Italy versus ≥58 % in other countries). We also observed a slightly lower proportion of parous women in the Netherlands and the United Kingdom. In the French cohort, fewer parous women had ever breastfed (72 % versus ≥81 % in other countries).

**Table 1:** Age standardized^a^ reproductive and lifestyle characteristics of the EPIC study population by country

	Total	Denmark	France	Germany	Greece	Italy	Norway	Spain	Sweden	The Netherlands	United Kingdom
No. of participants	322,972	27,887	66,878	26,766	13,893	30,008	35,215	23,828	19,930	26,223	52,344
Number of deaths	14,383	1,879	2,913	671	527	834	756	625	1,583	1,419	3,176
Mean (SD)											
Age at recruitment, years	50.3 (9.6)	56.2 (4.4)	52.1 (6.6)	48.1 (8.9)	51.6 (12.3)	50.0 (8.0)	47.6 (4.3)	47.5 (8.3)	54.9 (8.1)	50.2 (11.6)	47.1 (14.1)
Duration OC use, years^b,c^	7.7 (7.3)	9.4 (6.4)	7.4 (6.3)	12.4 (8.5)	2.5 (2.8)	4.1 (4.8)	5.0 (4.5)	3.7 (3.6)	9.7 (8.3)	9.9 (7.3)	7.6 (6.8)
Number of FTPs^c,d,e^	2.3 (1.0)	2.2 (0.6)	2.3 (0.9)	2.0 (0.9)	2.3 (1.0)	2.1 (0.9)	2.4 (0.8)	2.8 (1.3)	2.2 (0.9)	2.6 (1.2)	2.3 (1.0)
Age at first FTP^c,d^	24.9 (4.4)	23.8 (3.1)	24.9 (3.7)	24.2 (4.4)	24.1 (4.7)	25.8 (4.3)	24.0 (3.7)	24.9 (3.8)	24.6 (4.1)	25.4 (4.3)	25.6 (4.9)
Breastfeeding, months^c,f^	9.6 (10.6)	9.4 (6.3)	5.5 (5.2)	5.4 (6.4)	13.7 (16.3)	9.1 (8.5)	13.6 (9.5)	13.1 (12.8)	9.7 (7.3)	6.6 (6.9)	9.9 (12.9)
Age at menarche, years^c^	13.1 (1.5)	13.7 (1.2)	12.9 (1.3)	13.2 (1.5)	13.2 (1.6)	12.6 (1.5)	13.3 (1.1)	13.0 (1.6)	13.3 (1.5)	13.3 (1.6)	12.9 (1.6)
Age at menopause, years^c,g^	49.4 (4.4)	49.9 (3.9)	50.2 (4.3)	50.1 (3.2)	48.0 (4.7)	49.2 (4.1)	48.6 (3.3)	48.8 (4.4)	–	49.1 (4.5)	49.2 (4.6)
BMI, kg/m^2^	24.9 (4.4)	25.5 (3.2)	23.0 (3.2)	25.7 (4.6)	28.5 (5.2)	25.6 (4.3)	24.5 (3.1)	28.5 (4.6)	24.7 (4.2)	25.1 (4.2)	24.6 (4.2)
Percentages											
Ever use OCs^c^	59	58	60	80	11	40	60	36	61	76	68
Parous^c^	85	88	90	86	90	87	92	89	86	81	78
Ever breastfed^c,e^	85	93	72	84	88	84	94	89	95	81	83
Infertility^c,h^	5	–	6	1	4	6	–	4	–	3	5
Premenopausal	35	7	27	37	38	38	23	42	27	37	38
Postmenopausal	43	73	45	42	45	43	44	41	48	40	43
Surgical menopause^i^	3	5	3	3	6	4	0	6	0	2	4
Perimenopausal/unknown menopause	19	16	25	17	12	15	32	11	26	20	15
Hysterectomy^c,j^	11	14	10	16	9	9	5	10	–	16	16
Oophorectomy^c,j^	7	10	6	8	8	8	1	9	–	8	8
Current smoker^c,k^	20	31	9	18	19	26	32	15	25	29	11
Marital status: single	9	0	17	8	4	6	0	0	7	12	9
Ever married^l^	72	0	80	92	96	93	81	0	92	87	90
Marital status: unknown	19	100	4	0	0	1	19	100	0	0	0

BMI, Body mass index; FTP, Full term pregnancy; OCs, Oral contraceptives

^a^All variables (with the exception of age) were age standardized using 5-year age groups to the age distribution of the entire study population

^b^Among ever users of OCs

^c^Variables had missing data (≤4.2 % missing) with the exception of ever breastfed among parous (6.1 % missing), duration OC use in ever users (9.5 % missing), and age at natural menopause (26.3 % missing)

^d^FTPs includes live births and stillbirths

^e^Among parous women

^f^Among women with a FTP who had ever breastfed

^g^Postmenopausal women only; exclusions were participants from Sweden (no data for hysterectomy/oophorectomy), those who reported a surgical menopause (hysterectomy/oophorectomy), or if information on surgical menopause was not available

^h^Denmark, Sweden, Norway and the Bilthoven study centre were excluded since data were not available

^i^Defined as bilateral oophorectomy only

^j^Sweden was excluded from these comparisons because data were not available

^k^Current smoking included smoking of cigarettes, pipes, or cigars

^l^Ever married includes living together, divorced, separated, and widowed

### Association of reproductive factors with all-cause mortality

Parity was associated with a lower risk of all-cause mortality (parous versus nulliparous; HR = 0.80; CI, 0.76–0.84; Table 2). Having an early or late age at first FTP versus having a first FTP at age 26–30 was associated with a higher risk for all-cause mortality (first FTP ≤20: HR = 1.10; CI, 1.04–1.17; first FTP ≥31: HR = 1.08; CI, 1.01–1.16). Among parous women, ever versus never breastfeeding was inversely associated with risk of all-cause mortality (HR = 0.92; CI, 0.87–0.97). In contrast, there was no association with the number of FTPs or breastfeeding duration (*P* for trend ≥0.79).

**Table 2:** Association of reproductive characteristics with all-cause mortality in the EPIC study

Variable	Value	Cases / non-cases	Model 1 HR^a^ (95 % CI)	Model 2 HR^b^ (95 % CI)
Parous	No	2,220 / 45,746	1.00 (reference)	1.00 (reference)
	Yes	11,757 / 254,871	0.80 (0.76–0.83)	0.80 (0.76–0.84)
Number of FTPs^c^	1	2,307 / 45,881	1.00 (reference)	1.00 (reference)
	2	5,026 / 122,430	0.87 (0.83–0.91)	0.91 (0.86–0.96)
	3	2,529 / 56,316	0.83 (0.79–0.88)	0.87 (0.82–0.92)
	4	1,047 / 16,278	0.97 (0.90–1.04)	0.98 (0.91–1.06)
	5	350 / 4,448	0.97 (0.87–1.09)	0.97 (0.86–1.09)
	≥6	237 / 2,290	1.09 (0.95–1.25)	1.08 (0.94–1.24)
	*P* for trend^d^		0.91	0.79
Age at first FTP, years^c^	≤20	1,812 / 37,390	1.30 (1.23–1.38)	1.10 (1.04–1.17)
	21–23	3,121 / 68,203	1.13 (1.07–1.19)	1.05 (1.00–1.11)
	24–25	2,173 / 49,889	1.02 (0.97–1.08)	1.00 (0.95–1.06)
	26–30	3,287 / 72,649	1.00 (reference)	1.00 (reference)
	≥31	1,281 / 25,749	1.08 (1.01–1.15)	1.08 (1.01–1.16)
Breastfeeding^c^	Never	1,721 / 36,691	1.00 (reference)	1.00 (reference)
	Ever	9,148 / 202,910	0.88 (0.83–0.93)	0.92 (0.87–0.97)
Breastfeeding, months^e^	≤1	949 / 20,021	1.00 (reference)	1.00 (reference)
	>1 to ≤3	1,753 / 39,935	1.01 (0.93–1.09)	1.01 (0.93–1.09)
	>3 to ≤6	1,846 / 41,685	0.94 (0.87–1.02)	0.97 (0.89–1.05)
	>6 to ≤12	2,224 / 47,802	0.90 (0.83–0.98)	0.95 (0.88–1.03)
	>12 to ≤18	986 / 23,319	0.81 (0.74–0.89)	0.88 (0.80–0.97)
	>18	1,276 / 27,949	0.88 (0.80–0.96)	0.97 (0.89–1.07)
	*P* for trend^d^		0.013	0.85
Infertility^f^	No	8,958 / 199,770	1.00 (reference)	1.00 (reference)
	Yes	360 / 9,869	1.06 (0.95–1.18)	1.06 (0.95–1.18)
OC use in never or former	Never	6,379 / 100,292	1.00 (reference)	1.00 (reference)
smokers at baseline^g^	Ever	3,736 / 140,642	0.90 (0.85–0.94)	0.90 (0.86–0.95)
	Former^h^	3,475 / 122,414	0.89 (0.85–0.94)	0.90 (0.86–0.95)
	Current^h^	140 / 12,959	0.89 (0.74–1.07)	0.92 (0.76–1.11)
Duration OC use, years^g,i^	≤1	679 / 25,490	1.00 (reference)	1.00 (reference)
	>1 to <5	721 / 30,600	0.99 (0.89–1.10)	0.99 (0.89–1.11)
	5 to <10	705 / 30,859	0.95 (0.85–1.06)	0.96 (0.86–1.07)
	10 to <15	616 / 21,267	1.05 (0.94–1.18)	1.06 (0.94–1.18)
	≥15	526 / 18,583	1.02 (0.90–1.15)	1.02 (0.91–1.16)
	*P* for trend^d^		0.36	0.32
Age at menarche, years	<12	1,977 / 45,763	1.00 (reference)	1.00 (reference)
	12	2,714 / 65,654	0.92 (0.86–0.97)	0.95 (0.89–1.01)
	13	3,378 / 79,701	0.88 (0.84–0.94)	0.92 (0.87–0.98)
	14	3,224 / 66,159	0.89 (0.84–0.95)	0.93 (0.88–0.98)
	≥15	2,774 / 47,743	0.88 (0.82–0.93)	0.90 (0.85–0.96)
	*P* for trend^d^		0.004	0.038
Age at natural menopause, years^j^	≤45	1,080 / 12,399	1.15 (1.07–1.24)	1.09 (1.01–1.17)
	46–50	2,427 / 32,488	1.00 (reference)	1.00 (reference)
	51–52	1,064 / 14,537	0.88 (0.82–0.95)	0.91 (0.85–0.99)
	53–55	1,003 / 12,753	0.86 (0.80–0.93)	0.92 (0.85–0.99)
	>55	339 / 3,287	0.90 (0.80–1.01)	0.95 (0.85–1.07)
	*P* for trend^d^		<0.001	<0.001
Total ovulatory lifespan, years^k,l^	0–22	1,180 / 56,387	1.00 (reference)	1.00 (reference)
	22–29	1,621 / 53,629	0.96 (0.89–1.05)	0.99 (0.91–1.07)
	29–34	2,148 / 52,270	0.92 (0.84–0.99)	0.96 (0.89–1.04)
	≥34	3,115 / 51,923	0.89 (0.82–0.97)	0.98 (0.91–1.06)
	*P* for trend^d^		0.004	0.86
Oophorectomy^l^	No	11,167 / 266,535	1.00 (reference)	1.00 (reference)
	Yes	1,202 / 18,603	1.05 (0.98–1.11)	1.01 (0.93–1.10)
Hysterectomy^l^	No	10,606 / 257,298	1.00 (reference)	1.00 (reference)
	Yes	1,972 / 30,608	1.01 (0.96–1.06)	1.00 (0.94–1.05)

FTP, Full term pregnancy; OCs, Oral contraceptives

^a^Cox regression stratified by age (continuous) and study centre

^b^Cox regression adjusted for menopausal status (where applicable) (premenopausal [Ref], perimenopausal/unknown menopause, postmenopausal and surgical postmenopausal [bilateral oophorectomy]), body mass index (<23, 23–24.9 [Ref], 25–29.9, 30–39.9, 40+ kg/m^2^), physical activity index (inactive [Ref], moderately inactive, moderately active, active, unknown), education status (none/primary school [Ref], technical/professional school, secondary school/longer education including university, or unknown), smoking status/intensity (never [Ref], current 1–15 cigarettes/day, current 16–25 cigarettes/day, current 26+ cigarettes/day, former quit ≤10 years, former quit 11–20 years, former quit >20 years, current pipe/cigar/occasional smoker, current/former missing timing, unknown), smoking duration (<10 [Ref], 10 to <20, 20 to <30, 30 to <40, 40+ years, unknown), and stratified by age and study centre

^c^Restricted to parous women. A FTP was defined as live births and stillbirths

^d^ *P* for trend values are based on continuous variables: the number of FTPs was modelled as 1 to ≥10; age at menarche as ≤8, 9–19, ≥20; age at menopause as <35, 35–60, >60

^e^Among parous women who had ever breastfed (sum of all FTPs)

^f^Denmark, Sweden, Norway, and the Bilthoven (Netherlands) study centre were excluded since data were not available

^g^Restricted to participants who were never or former smokers, therefore models were adjusted for the same factors^b^ and a modified smoking status/intensity variable (never [Ref], former quit ≤10 years, former quit 11–20 years, former quit >20 years, former missing quit years, occasional smoker)

^h^Does not add up to the total number of OC users because 3.7 % of OC users were missing their timing of use

^i^ Among ever users of OCs

^j^Among postmenopausal women; excluded participants from Sweden (no data for hysterectomy/oophorectomy), those who reported a surgical menopause (hysterectomy and/or unilateral/bilateral oophorectomy), or if information on surgical menopause was missing

^k^The total ovulatory lifespan is the difference between the age at natural menopause and the age at menarche for postmenopausal women, or the difference between the age at recruitment and the age at menarche for premenopausal/perimenopausal/unknown menopausal status women, minus the amount of time that a woman was pregnant (number of full term pregnancies × nine months) and/or used OCs

^l^Sweden was excluded from these comparisons (no data for hysterectomy/oophorectomy), therefore age at natural menopause could not be determined

Having a late versus early age at menarche was associated with a lower risk of all-cause mortality; women who underwent menarche at age ≥15 years compared with age <12 had a 10 % reduction in risk of death (HR = 0.90; CI, 0.85–0.96) and there was an inverse trend across age at menarche categories (*P* for trend = 0.038). Having an early age at natural menopause was associated with a higher risk of all-cause mortality (≤45 years versus 46–50; HR = 1.09; CI, 1.01–1.17), while overall there was a suggestion of lower risk with a later age at menopause (*P* for trend <0.001). There was no association between infertility, total ovulatory years, oophorectomy, or hysterectomy with risk of all-cause mortality.

In stratified analyses, an inverse association between ever versus never use of OCs and risk of all-cause mortality was observed only in never/former smokers (HR = 0.90; CI, 0.86–0.95; n = 3,736 deaths were observed among ever users of OCs), but not current smokers (OC ever versus never use; HR = 0.98; CI, 0.91–1.05; n = 2,076 deaths occurred in ever users of OCs, *P* for interaction = 0.0021); analyses of OCs and mortality risk were therefore restricted to participants who were never/former smokers. There was a similar lower risk of all-cause mortality in former users and current users of OCs at the study baseline, although the finding for current users was non-significant possibly due to the small number of deaths (n = 140) in this category. The lowered risk of all-cause mortality associated with ever use of OCs was observed across all categories grouped by calendar year of first use (before 1970: HR = 0.89; CI, 0.84–0.95; 1970s: HR = 0.89; CI, 0.83–0.95; 1980s and later: HR = 0.84; CI, 0.71–0.99; data not shown). In contrast, there was no association with the duration of OC use among ever users of OCs irrespective of smoking status (*P* for interaction = 0.283 for never/former and current smokers).

The inverse association between having a late age at menarche (age ≥15 years versus <12) and risk of all-cause mortality was only observed in women with a BMI ≥25 kg/m^2^ (HR = 0.82; CI, 0.75–0.89; *P* for trend = 0.0034). In contrast, there was no association for participants with a BMI <25 kg/m^2^ (HR = 0.98; CI, 0.90–1.07; *P* for trend = 0.58; *P* for interaction = 0.016). Results from the stratified analyses are detailed in Additional file 2. The higher incidence of all-cause mortality among women with an early age at menopause (menopause age ≤45 years versus 46–50) was restricted to former and current smokers (HR = 1.22; CI, 1.10–1.35; *P* for trend <0.001), while there was no association in never smokers (HR = 1.02; CI, 0.91–1.13; *P* for trend = 0.35; *P* for interaction = 0.012). There was no difference in the risk associations for all-cause mortality in analyses of parity when stratifying by marital status, for the number of FTPs according to BMI, or with breastfeeding duration by the number of FTPs (*P* for interaction ≥0.34). In sensitivity analyses, we observed similar results for all-cause mortality analyses when restricting analyses to postmenopausal women only, or when participants who reported prevalent conditions (diabetes, heart conditions, or cancer) were not excluded (data not shown). In sensitivity analyses of age at menopause without excluding women who reported a surgically-induced menopause in relation to breast cancer mortality, we observed similar results to those reported for age at natural menopause (data not shown).

### Cancer mortality

Risk of total cancer mortality was lower in parous versus nulliparous women (HR = 0.87; CI, 0.80–0.93) and with a later age at menarche (age ≥15 years versus <12; HR = 0.89; CI, 0.81–0.98; *P* for trend = 0.042; Table 3). Among parous women, having two or three FTPs versus one was associated with a lower risk of total cancer mortality (e.g. 3 FTPs versus 1; HR = 0.89; CI, 0.81–0.97), but having more than three FTPs versus one was not associated with risk. Among never/former smokers, we observed a lower risk of total cancer mortality in ever versus never users of OCs (HR = 0.91; CI, 0.85–0.98), while there was no association with OC use in participants who were current smokers at baseline (ever versus never users of OCs; HR = 1.00; CI, 0.90–1.12). In analyses of breast cancer mortality, there was a strong decreased risk in parous versus nulliparous women (HR = 0.70; CI, 0.58–0.85) and a trend of lower mortality risk with a higher number of FTPs (*P* for trend = 0.012). There was no association with other reproductive characteristics although the number of breast cancer deaths (n = 749) was limited.

**Table 3:** Association of reproductive characteristics with total cancer and breast cancer mortality in the EPIC study

		Total cancer (n = 5,938)		Breast cancer (n = 749)	
ICD-10 code		C00–D48		C50	
Variable	Value	Cases/ non-cases	HR^a^ (95 % CI)	Cases / non-cases	HR^a^ (95 % CI)
Parous	Yes	4,973 / 261,634	0.87 (0.80–0.93)	598 / 266,009	0.70 (0.58–0.85)
Number of FTPs^b,c^	1	944 / 47,240	1.00 (reference)	135 / 48,049	1.00 (reference)
	2	2,164 / 125,284	0.91 (0.84–0.98)	268 / 127,180	0.74 (0.60–0.92)
	3	1,069 / 57,773	0.89 (0.81–0.97)	119 / 58,723	0.70 (0.54–0.90)
	4	442 / 16,880	1.05 (0.93–1.18)	55 / 24,591	0.68 (0.49–0.95)
	5	118 / 4,680	0.86 (0.71–1.05)		
	≥6	88 / 2,438	1.11 (0.89–1.39)		
	*P* for trend^d^		0.85		0.012
Age at first FTP, years^b^	≤20	781 / 38,420	1.03 (0.94–1.14)	90 / 39,111	0.89 (0.68–1.16)
	21–23	1,267 / 70,055	0.97 (0.90–1.05)	135 / 71,187	0.79 (0.63–0.99)
	24–25	915 / 51,141	0.95 (0.87–1.03)	114 / 51,942	0.91 (0.72–1.15)
	26–30	1446 / 74,481	1.00 (reference)	187 / 75,740	1.00 (reference)
	≥31	531 / 26,496	1.06 (0.96–1.17)	70 / 26,957	1.05 (0.79–1.39)
Breastfeeding^e^	Ever	3,964 / 208,077	0.99 (0.91–1.08)	484 / 211,557	1.01 (0.79–1.29)
Breastfeeding, months^f^	≤1	399 / 20,571	1.00 (reference)	58 / 20,912	1.00 (reference)
	>1 to ≤3	768 / 40,917	0.96 (0.85–1.09)	102 / 41,583	0.87 (0.62–1.21)
	>3 to ≤6	783 / 42,744	0.92 (0.81–1.04)	82 / 43,445	0.68 (0.48–0.96)
	>6 to ≤12	965 / 49,056	0.93 (0.83–1.05)	101 / 49,920	0.69 (0.49–0.97)
	>12 to ≤18	434 / 23,868	0.87 (0.76–1.00)	63 / 24,239	0.88 (0.60–1.27)
	>18	579 / 28,644	1.04 (0.90–1.19)	74 / 29,149	0.94 (0.65–1.37)
	*P* for trend^d^		0.23		0.35
OC use in never or former	Never	2,376 / 104,288	1.00 (reference)	257 / 106,407	1.00 (reference)
smokers at baseline^g^	Ever	1,775 / 142,587	0.91 (0.85–0.98)	313 / 144,049	1.11 (0.91–1.35)
	Former^h^	1,656 / 124,219	0.91 (0.85–0.98)	291 / 125,584	1.12 (0.92–1.36)
	Current^h^	78 / 13,019	0.93 (0.73–1.20)	18 / 13,079	0.87 (0.50–1.50)
Duration of OC use, years^g,i^	≤1	325 / 25,843	1.00 (reference)	53 / 26,115	1.00 (reference)
	>1 to <5	340 / 30,979	0.96 (0.82–1.12)	64 / 31,255	1.00 (0.69–1.45)
	5 to <10	372 / 31,188	1.06 (0.91–1.23)	59 / 31,501	0.94 (0.64–1.37)
	10 to <15	305 / 21,576	1.09 (0.92–1.28)	63 / 21,818	1.33 (0.91–1.94)
	≥15	277 / 18,825	1.07 (0.90–1.27)	53 / 19,049	1.22 (0.81–1.83)
	*P* for trend^d^		0.195		0.106
Age at menarche, years	<12	811 / 46,926	1.00 (reference)	98 / 47,639	1.00 (reference)
	12	1,147 / 67,220	0.97 (0.89–1.06)	162 / 68,205	1.17 (0.91–1.51)
	13	1,380 / 81,686	0.91 (0.83–0.99)	164 / 82,902	0.93 (0.72–1.20)
	14	1,365 / 68,009	0.95 (0.87–1.04)	198 / 69,176	1.27 (0.99–1.63)
	≥15	1,125 / 49,388	0.89 (0.81–0.98)	114 / 50,399	0.89 (0.67–1.18)
	*P* for trend^d^		0.042		0.35
Age at natural menopause, years^j^	≤45	371 / 13,105	0.97 (0.86–1.10)	30 / 13,446	0.68 (0.45–1.03)
	46–50	988 / 33,921	1.00 (reference)	120 / 34,789	1.00 (reference)
	51–52	448 / 15,151	0.98 (0.88–1.10)	43 / 15,556	0.80 (0.56–1.14)
	53–55	448 / 13,307	1.08 (0.96–1.21)	53 / 13,702	1.03 (0.73–1.45)
	>55	141 / 3,483	1.12 (0.93–1.34)	13 / 3,611	0.87 (0.47–1.61)
	*P* for trend^d^		0.080		0.37

FTP, Full term pregnancy; OCs, Oral contraceptives; ICD-10, International Statistical Classification of Diseases Injuries and Causes of Death (10^th^ revision)

^a^Cox regression adjusted for menopausal status (where applicable) (premenopausal [Ref], perimenopausal/unknown menopause, postmenopausal and surgical postmenopausal (bilateral oophorectomy)), body mass index (<23, 23–24.9 [Ref], 25–29.9, 30–39.9, 40+ kg/m^2^), physical activity index (inactive [Ref], moderately inactive, moderately active, active, unknown), education status (none/primary school [Ref], technical/professional school, secondary school/longer education including university, or unknown), smoking status/intensity (never [Ref], current 1–15 cigarettes/day, current 16–25 cigarettes/day, current 26+ cigarettes/day, former quit ≤10 years, former quit 11–20 years, former quit >20 years, current pipe/cigar/occasional smoker, current/former missing timing, unknown), smoking duration (<10 [Ref], 10 to <20, 20 to <30, 30 to <40, 40+ years, unknown), and stratified by age (continuous) and study centre

^b^FTP includes live births and stillbirths

^c^For breast cancer the top categories were combined as indicated due to small numbers

^d^ *P* for trend values are based on continuous variables: the number of FTPs was modelled as 1 to ≥10; age at menarche as ≤8, 9–19, ≥20; age at menopause as <35, 35–60, >60

^e^Among parous women

^f^Among parous women who had ever breastfed (sum of all FTPs)

^g^Restricted to participants who were never or former smokers, therefore models were adjusted for the same factors^a^ and a modified smoking status/intensity variable (never [Ref], former quit ≤10 years, former quit 11–20 years, former quit >20 years, former missing quit years, occasional smoker)

^h^Does not add up to the total number of OC users because 3.7 % of OC users were missing their timing of use

^i^Among ever users of OCs

^j^Among postmenopausal women; exclusions included participants from Sweden (no data for hysterectomy/oophorectomy), those who had a surgical menopause (hysterectomy and/or oophorectomy), or if information on surgical menopause was missing

### Circulatory disease mortality

Reproductive characteristics that were inversely associated with risk of death from circulatory disease included parity (parous versus nulliparous; HR = 0.86; CI, 0.76–0.96), breastfeeding (ever versus never breastfed; HR = 0.80; CI, 0.70–0.91), and a late age at menarche (≥15 years versus <12; HR = 0.83; CI, 0.72–0.96; Table 4). Never/former smokers at baseline who had ever versus never used OCs had a lower risk of circulatory disease death (HR = 0.85; CI, 0.75–0.97). In contrast, among current smokers at baseline, there was no association with ever use of OCs (HR = 0.98; CI, 0.81–1.17). There was a small but non-significant increase in risk of circulatory disease death for current OC users as compared with never users of OCs at baseline (HR = 1.48; CI, 0.90–2.43; based on n = 22 deaths). In sub-analyses of ischaemic heart disease mortality, we observed strong inverse associations with parity (parous versus nulliparous women), breastfeeding (ever versus never breastfed), and a suggestive inverse association with a late versus early age at natural menopause. In analyses of death from cerebrovascular disease, there were non-significant inverse associations with a late versus early age at menarche, and in ever versus never users of OCs (former/never smokers only). In contrast, non-smoking participants who reported current OC use at the study baseline had a higher risk of cerebrovascular disease death (HR = 2.62; CI, 1.30–5.26), but this finding was based on a small number (n = 13) of deaths; there were too few current OC users who were also current smokers at the study baseline to evaluate this subgroup.

**Table 4:** Association of reproductive characteristics with mortality from circulatory diseases in the EPIC study

		Circulatory disease (n = 2,404)		Cerebrovascular disease (n = 808)		Ischaemic heart disease (n = 732)	
ICD-10 codes		I00–I99		I60–I69		I20–I25	
Variable	Value	Cases / non-cases	HR^a^ (95 % CI)	Cases / non-cases	HR^a^ (95 % CI)	Cases / non-cases	HR^a^ (95 % CI)
Parous	Yes	1,970 / 264,637	0.86 (0.76–0.96)	655 / 265,952	0.93 (0.76–1.13)	598 / 266,009	0.79 (0.65-0.97)
Number of FTPs^b^	1	374 / 47,810	1.00 (reference)	114 / 48,070	1.00 (reference)	117 / 48,067	1.00 (reference)
	2	799 / 126,649	0.92 (0.81–1.04)	281 / 127,167	1.06 (0.85–1.32)	240 / 127,208	0.91 (0.73-1.14)
	3	431 / 58,411	0.88 (0.76–1.02)	135 / 58,707	0.90 (0.70–1.17)	139 / 58,703	0.93 (0.73-1.20)
	4	198 / 17,124	0.95 (0.79–1.13)	66 / 17,256	1.03 (0.75–1.41)	48 / 17,274	0.74 (0.52-1.05)
	5	63 / 4,735	0.85 (0.65–1.12)	21 / 4,777	0.92 (0.57–1.48)	18 / 4,780	0.78 (0.47-1.29)
	≥6	62 / 2,464	1.13 (0.85–1.50)	23 / 2,503	1.49 (0.93–2.39)	19 / 2,507	1.12 (0.67-1.88)
	*P* for trend^c^		0.85		0.42		0.29
Age at first FTP, years^d^	≤20	304 / 38,897	1.15 (0.99–1.34)	97 / 39,104	1.14 (0.86–1.50)	105 / 39,096	1.14 (0.86-1.51)
	21–23	497 / 70,825	1.11 (0.98–1.26)	150 / 71,172	0.96 (0.76–1.23)	140 / 71,182	0.96 (0.74-1.24)
	24–25	359 / 51,697	1.05 (0.92–1.20)	122 / 51,934	1.00 (reference)	106 / 51,950	1.00 (reference)
	26–30	557 / 75,370	1.00 (reference)	199 / 75,728	0.97 (0.78–1.23)	166 / 75,761	0.99 (0.77-1.26)
	≥31	237 / 26,790	1.06 (0.90–1.23)	79 / 26,948	0.90 (0.67–1.21)	74 / 26,953	1.10 (0.81-1.49)
Breastfeeding^d^	Ever	1,534 / 210,507	0.80 (0.70–0.91)	526 / 211,515	0.94 (0.74–1.21)	445 / 211,596	0.69 (0.54-0.87)
Breastfeeding, months^e^	≤1	135 / 20,835	1.00 (reference)	38 / 20,932	1.00 (reference)	41 / 20,929	1.00 (reference)
	>1 to ≤3	282 / 41,403	1.16 (0.94–1.43)	101 / 41,584	1.47 (1.00–2.14)	86 / 41,599	1.22 (0.83-1.79)
	>3 to ≤6	276 / 43,251	1.05 (0.85–1.30)	93 / 43,434	1.20 (0.82–1.77)	91 / 43,436	1.22 (0.83-1.79)
	>6 to ≤12	359 / 49,662	1.02 (0.83–1.26)	115 / 49,906	1.10 (0.75–1.60)	102 / 49,919	0.99 (0.68-1.45)
	>12 to ≤18	184 / 24,118	0.98 (0.78–1.23)	69 / 24,233	1.18 (0.78–1.77)	51 / 24,251	0.94 (0.61-1.44)
	>18	287 / 28,936	1.04 (0.83–1.31)	110 / 29,113	1.28 (0.85–1.90)	71 / 29,152	0.92 (0.60-1.40)
	*P* for trend^c^		0.48		0.076		0.153
OC use in never or former	Never	1,274 / 105,390	1.00 (reference)	467 / 106,197	1.00 (reference)	352 / 106,312	1.00 (reference)
smokers at baseline^f^	Ever	427 / 143,935	0.85 (0.75–0.97)	144 / 144,218	0.80 (0.64–1.01)	102 / 144,260	0.87 (0.67-1.12)
	Former^g^	397 / 125,478	0.84 (0.74–0.96)	129 / 125,746	0.78 (0.62–0.98)	100 / 125,775	0.87 (0.67-1.13)
	Current^g,h^	22 / 13,075	1.48 (0.90–2.43)	13 / 13,084	2.62 (1.30–5.26)	2 / 13,095	-
Duration of OC use, years^f,i^	≤1	72 / 26,096	1.00 (reference)	23 / 26,145	1.00 (reference)	21 / 26,147	1.00 (reference)
	>1 to <5	72 / 31,247	0.95 (0.68–1.33)	23 / 31,296	0.95 (0.52–1.73)	21 / 31,298	0.97 (0.52-1.83)
	5 to <10	80 / 31,480	0.98 (0.70–1.36)	34 / 31,526	1.40 (0.80–2.44)	14 / 31,546	0.58 (0.29-1.16)
	10 to <15	84 / 21,797	1.22 (0.88–1.70)	22 / 21,859	1.08 (0.59–2.00)	22 / 21,859	1.09 (0.58-2.05)
	≥15	70 / 19,032	1.14 (0.80–1.62)	30 / 19,072	1.63 (0.91–2.94)	15 / 19,087	0.96 (0.48-1.94)
	*P* for trend^c^		0.38		0.177		0.91
Age at menarche, years	<12	325 / 47,412	1.00 (reference)	105 / 47,632	1.00 (reference)	107 / 47,630	1.00 (reference)
	12	411 / 67,956	0.84 (0.73–0.98)	132 / 68,235	0.80 (0.62–1.04)	123 / 68,244	0.80 (0.62-1.04)
	13	555 / 82,511	0.89 (0.77–1.02)	186 / 82,880	0.86 (0.67–1.09)	165 / 82,901	0.81 (0.63-1.03)
	14	559 / 68,815	0.91 (0.79–1.05)	189 / 69,185	0.88 (0.69–1.12)	164 / 69,210	0.80 (0.62-1.02)
	≥15	494 / 50,019	0.83 (0.72–0.96)	165 / 50,348	0.79 (0.61–1.02)	160 / 50,353	0.80 (0.62-1.03)
	*P* for trend^c^		0.47		0.51		0.30
Age at natural menopause, years^j^	≤45	251 / 13,225	1.07 (0.91–1.25)	78 / 13,398	1.00 (0.75–1.32)	85 / 13,391	1.14 (0.86-1.50)
	46–50	499 / 34,410	1.00 (reference)	163 / 34,746	1.00 (reference)	157 / 34,752	1.00 (reference)
	51–52	189 / 15,410	0.79 (0.67–0.94)	68 / 15,531	0.83 (0.62–1.12)	43 / 15,556	0.58 (0.41-0.82)
	53–55	166 / 13,589	0.75 (0.63–0.90)	54 / 13,701	0.71 (0.52–0.97)	45 / 13,710	0.67 (0.48-0.94)
	>55	64 / 3,560	0.85 (0.65–1.11)	22 / 3,602	0.81 (0.51–1.28)	17 / 3,607	0.71 (0.42-1.18)
	*P* for trend^c^		<0.001		0.088		<0.001

FTP, Full term pregnancy; OCs, Oral contraceptives; ICD-10, International Statistical Classification of Diseases Injuries and Causes of Death (10^th^ revision)

^a^Cox regression adjusted for menopausal status (where applicable) (premenopausal [Ref], perimenopausal/unknown menopause, postmenopausal and surgical postmenopausal (bilateral oophorectomy)), body mass index (<23, 23–24.9 [Ref], 25–29.9, 30–39.9, 40+ kg/m^2^), physical activity index (inactive [Ref], moderately inactive, moderately active, active, unknown), education status (none/primary school [Ref], technical/professional school, secondary school/longer education including university, or unknown), smoking status and intensity (never [Ref], current 1–15 cigarettes/day, current 16–25 cigarettes/day, current 26+ cigarettes/day, former quit ≤10 years, former quit 11–20 years, former quit >20 years, current pipe/cigar/occasional smoker, current/former missing timing, unknown), smoking duration (<10 [Ref], 10 to <20, 20 to <30, 30 to <40, 40+ years, unknown), and stratified by age (continuous) and study centre

^b^FTP includes live births and stillbirths

^c^ *P* for trend-values are based on continuous variables: number of FTPs (1 to ≥10); age at menarche (≤8, 9–19, ≥20); age at menopause (<35, 35–60, >60)

^d^Among parous women

^e^Among parous women who had ever breastfed (sum of all FTPs)

^f^Restricted to participants who were never or former smokers, therefore models were adjusted for the same factors^a^ and a modified smoking status/intensity variable (never [Ref], former quit ≤10 years, former quit 11–20 years, former quit >20 years, former missing quit years, occasional smoker)

^g^Does not add up to the total number of OC users because 3.7 % of OC users were missing their timing of use

^h^Of 22 total circulatory disease deaths, 13 were due to cerebrovascular disease, 2 to ischaemic heart disease, and 7 to other circulatory-related causes (n = 2 ‘Pulmonary embolism without mention of acute cor pulmonale’, and one death for each cause ‘Essential (primary) hypertension’, ‘Other specified pulmonary heart diseases’, ‘Cardiac arrest, unspecified’, ‘Atrial fibrillation and flutter’ and ‘Phlebitis and thrombophlebitis of other deep vessels of lower extremities’)

^i^Among ever users of OCs

^j^Among postmenopausal women; exclusions included participants from Sweden (no data for hysterectomy/oophorectomy), those who had a surgical menopause (hysterectomy and/or unilateral/bilateral oophorectomy), or if information on surgical menopause was missing

## Discussion

In a large and comprehensive prospective study representing data from 10 European countries, we observed that, after controlling for factors known to influence mortality risk (such as BMI, smoking habits and physical activity), childbirth, breastfeeding among parous women, ever use of OCs among non-smokers, a later age at menopause, and a later age at menarche were associated with a significantly lower risk of all-cause mortality. Most of these associations were also apparent when we considered cause-specific deaths from total cancer and ischaemic heart disease. Importantly, these reproductive factors are common exposures and with a better understanding of how these factors may influence long-term health this information may assist in the development of new clinical strategies for the improvement of women’s health.

The inverse association for parous as compared with nulliparous women with risk of all-cause, total cancer, breast cancer, and ischaemic heart disease mortality is consistent with previous studies [13–17, 43, 44]. It is possible that other underlying factors may explain the association between parous versus nulliparous women; for example, some women may be nulliparous because they were chronically unwell. However, our data did not suggest that nulliparous women had poorer health as their BMI, physical activity levels, and smoking status were similar to parous women. Notable differences were that a higher proportion of nulliparous women had obtained a higher education level and fewer nulliparous women had ever married. Among parous women, we observed a trend of lower risk for breast cancer mortality with an increasing number of FTPs. In contrast, the number of FTPs was not associated with other mortality outcomes. It has been suggested that having ≥4 births may increase a mother’s risk of circulatory disease mortality [21], possibly by inducing hypertensive changes [45] and/or by increasing their body weight [46]. However, in a study of highly parous women in Northern Finland, only women with ≥10 births (versus 2–4) had a higher risk of mortality from haemorrhagic stroke [20]. Consistent with previous studies [8, 23], we observed that parous women who had ever breastfed had a lower risk of mortality from all-causes and ischaemic heart disease. Prior studies have observed that women who never breastfed [47] and/or had a shorter lactation period [22, 48] had a higher risk to develop hypertension. Similar to prior reports [4–6, 8], we observed that a later age at menarche was associated with a lower risk of all-cause, total cancer, and circulatory mortality. Having an earlier age at menarche has been associated with elevated blood pressure and glucose intolerance [49], increased body fat in early adulthood [50], or obesity in adulthood [51], all of which could explain the possible link between the age at menarche and risk of mortality outcomes later in life. We also observed that an early age at menopause was associated with a higher risk of all-cause mortality, but this association was attenuated and non-significant in never smokers, which suggests that there may be residual confounding by factors that influence the age of menopause [52].

In analyses of OC use, we observed a lower risk for all-cause, total cancer, and circulatory disease mortality with ever versus never use of OCs among non-smokers; this finding is consistent with two studies [11, 12], but not others [8–10] that observed no association between OC use and mortality risk. In analyses of cerebrovascular disease mortality among non-smokers, we observed a non-significant lower risk of death in ever versus never users of OCs, while there was a higher risk of death among participants who reported current OC use at the study baseline although the latter finding was based on only 13 deaths. These results for current OC use contrast with previous reports of no association with risk of cerebrovascular disease death among current or recent users of OCs (use within <5 years) from the Nurses’ Health Study [9] and the Royal College of General Practitioners’ Oral Contraception Study [11]. In the current study, the participant’s mean age at recruitment was 50 years and most of the OC use reported at the study baseline referred to former use that likely occurred at least 10–15 years ago; these findings should be interpreted in this context. We also explored the relationship between OC use and mortality separately for never/former and current smokers; however, smoking history was based on information at the study baseline and therefore may not reflect the smoking habits at the time that OCs were used. It has been reported that smoking 15+ cigarettes/day doubles the risk of all-cause mortality [12] and our results suggested that the possible benefits of OC use may not outweigh the harmful effects of smoking [53].

Together, these results highlight the possibility that hormonal mechanisms may explain the link between parity, breastfeeding, OC use, and a later age at menarche with a lower mortality risk. A shared mechanism for breastfeeding and OC use is that both may reduce endogenous estradiol production [54, 55]. A study in Finnish girls observed that having a later age at menarche was associated with lower estrogen levels [56, 57], but this result was not confirmed in studies of adult women [58, 59]. Although pregnancy raises serum estrogen levels, this is accompanied by elevated progesterone levels which may offset the proliferative effects of estrogen [60]. In contrast, breastfeeding and OC use reduce endogenous progesterone synthesis [55, 61]. Both parity and OC use would lower gonadotropin levels, specifically luteinizing hormone and follicle-stimulating hormone [55, 62], and girls with a late age at menarche also had reduced follicle-stimulating hormone levels [56]. On the other hand, breastfeeding lowers luteinizing hormone but increases follicle-stimulating hormone [54]. Our epidemiologic findings are not consistent with an androgen-related mechanism because parity is expected to increase, and OC use decrease, androgen and specifically testosterone levels [60, 63], while inconsistent results have been reported regarding the association between the age at menarche and androgen levels in postmenopausal women [64, 65]. Importantly, since most of these hormonal changes were measured at or near the time that these reproductive events occurred, additional studies are needed to evaluate how these reproductive characteristics may influence a woman’s long-term hormonal profile in order to highlight potential mechanisms that may explain the strong inverse associations between parity, breastfeeding, OC use, and a delayed age at menarche with risk of mortality that were observed in the current study.

Possible study limitations include the use of a single assessment of reproductive exposures at the study baseline; however, it is unlikely that reproductive characteristics would change particularly among postmenopausal women and, indeed, we observed similar results when analyses were restricted to the subgroup of postmenopausal women. Reproductive events that occurred many years previously may be subject to recall issues which could attenuate risk estimates towards the null; however, since we observed similar results when restricting to women who were postmenopausal at recruitment this suggests that this was not a major issue in this study. Although we accounted for important potential confounding variables such as BMI, smoking status, and education level in the analysis, we cannot exclude the possibility that other unmeasured factors, such as underlying differences in social class, may explain the observed associations. Another possible limitation is that the EPIC participants are not representative of the general population and they may have different distributions of risk factors, such as smoking and BMI, which may limit the generalizability of these findings. However, in support of our conclusions, many results were consistent with previous smaller-scale studies. Finally, advantages of this investigation include the representation of findings across 10 European countries and the near complete follow-up for vital status.

## Conclusions

This analysis of >320,000 European women highlighted several reproductive characteristics, including childbirth, breastfeeding, later age at menarche, and use of OCs in non-smokers, that may lower the risk of all-cause mortality. Further studies are needed to confirm these findings and to clarify the mechanisms that link these reproductive exposures to mortality risk. With a better understanding of the impact of reproductive characteristics on mortality risk, these data may be used to assist in the development of clinical strategies to improve the long-term health of women.

## Additional files

Additional file 1:
**List of all of the local ethics committees for the EPIC study.** (XLSX 19 kb) Additional file 2:
**Supplemental tables that are referred to in the manuscript text.** (DOCX 32 kb)

## Abbreviations

BMI: body mass index
CI: 95 % Confidence interval
EPIC: European Investigation into Cancer and Nutrition
FTP: full term pregnancy
HR: Hazard ratio
OCs: Oral contraceptives
