# Supplementary material for: Reproductive factors and risk of mortality in the European Prospective Investigation into Cancer and Nutrition; a cohort study
Source: BMC Med. 2015 Oct 30;13:252. doi: 10.1186/s12916-015-0484-3 (PMC4627614; doi:10.1186/s12916-015-0484-3)
Supplement: Additional file 2: — Supplemental tables that are referred to in the manuscript text. (DOCX 32 kb) [file 12916_2015_484_MOESM2_ESM.docx]

Merritt et al. 2015

Manuscript: “Reproductive Factors and Risk of Mortality in the European Prospective Investigation into Cancer and Nutrition; a Cohort Study”

This file includes supplemental tables that are referred to in the manuscript text.

**Table S1**. Association of age at menarche with all-cause mortality in the EPIC study after stratifying by BMI

|  |  | **BMI < 25 kg/m^2^** | | **BMI ≥ 25 kg/m^2^** | |
| --- | --- | --- | --- | --- | --- |
|  |  | **Cases/non-** | **Model HR^*^** | **Cases/non-** | **Model HR^*^** |
|  | **Value** | **cases** | **(95% CI)** | **cases** | **(95% CI)** |
| **Age at** | <12 | 841/23740 | 1.00 (Ref) | 1136/22023 | 1.00 (Ref) |
| **menarche, y** | 12 | 1399/37925 | 1.02 (0.93-1.11) | 1315/27729 | 0.89 (0.82-0.96) |
|  | 13 | 1879/48973 | 1.00 (0.92-1.09) | 1499/30728 | 0.84 (0.78-0.91) |
|  | 14 | 1754/41335 | 0.98 (0.90-1.06) | 1470/24824 | 0.88 (0.81-0.96) |
|  | ≥15 | 1559/29576 | 0.98 (0.90-1.07) | 1215/18167 | 0.82 (0.75-0.89) |
|  | P-trend^†^ |  | 0.58 |  | 0.0034 |
|  | P-int^‡^ | 0.016 |  |  |  |

Abbreviations: y, years.

^*^ Cox regression adjusted for menopausal status (premenopausal [Ref], perimenopausal/unknown menopause, postmenopausal and surgical postmenopausal [bilateral oophorectomy]), physical activity index (inactive [Ref], moderately inactive, moderately active, active, unknown), education status (none/primary school [Ref], technical/professional school, secondary school/longer education including university, or unknown), smoking status/intensity (never [Ref], current 1-15 cigarettes/day, current 16-25 cigarettes/day, current 26+ cigarettes/day, former quit ≤10 years, former quit 11-20 years, former quit >20 years, current pipe/cigar/occasional smoker, current/former missing timing, unknown), smoking duration (<10 [Ref], 10-<20, 20-<30, 30-<40, 40+ years, unknown), and stratified by age and study center..

^†^ P for trend values are based on continuous variables: age at menarche was modeled as ≤8,9-19,≥20.

^‡^ P for interaction value was calculated using a likelihood ratio test to compare multivariate models with and without multiplicative interaction terms between categories of age at menarche and BMI.

**Table S2**. Association of age at menopause with all-cause mortality in the EPIC study after stratifying by smoking status

|  |  | **Never smoker** | | **Former/current smoker** | |
| --- | --- | --- | --- | --- | --- |
|  |  | **Cases/** | **Model HR**^*^ | **Cases/** | **Model HR**^*^ |
|  | **Value** | **non-cases** | **(95% CI)** | **non-cases** | **(95% CI)** |
| **Age natural menopause, y**^†^ | ≤45 | 504/7155 | 1.02 (0.91-1.13) | 565/5064 | 1.22 (1.10-1.35) |
|  | 46-50 | 1199/19064 | 1.00 (Ref) | 1211/12977 | 1.00 (Ref) |
|  | 51-52 | 595/9205 | 0.94 (0.85-1.04) | 456/5128 | 0.87 (0.78-0.97) |
|  | 53-55 | 567/8597 | 0.88 (0.80-0.98) | 422/3993 | 0.92 (0.82-1.03) |
|  | >55 | 209/2205 | 1.02 (0.88-1.19) | 127/1045 | 0.85 (0.70-1.03) |
|  | P-trend^‡^ |  | 0.35 |  | <0.001 |
|  | P-int^§^ | 0.012 |  |  |  |

Abbreviations: y, years.

^*^ Cox regression adjusted for body mass index (<23, 23-24.9 [Ref], 25-29.9, 30-39.9, 40+ kg/m2), physical activity index (inactive [Ref], moderately inactive, moderately active, active, unknown), education status (none/primary school [Ref], technical/professional school, secondary school/longer education including university, or unknown), and stratified by age and study center.

^†^ Sweden was excluded from these comparisons (no data for hysterectomy/oophorectomy), therefore age at natural menopause could not be determined.

^‡^ P for trend values are based on continuous variables: age at menopause was modeled as <35,35-60,>60 years.

^§^ P for interaction value was calculated using a likelihood ratio test to compare multivariate models with and without multiplicative interaction terms between categories of age at menopause and smoking status.

**Table S3**. Association of parity with all-cause mortality in the EPIC study after stratifying by marital status**^*^**

|  | **Never married** | | **Ever married**^†^ | |
| --- | --- | --- | --- | --- |
|  | **Cases/** | **Model HR**^‡^ | **Cases/** | **Model HR**^‡^ |
| **Value** | **non-cases** | **(95% CI)** | **non-cases** | **(95% CI)** |
| Nulliparous | 819/17560 | 1.00 (Ref) | 970/21105 | 1.00 (Ref) |
| Parous | 477/8699 | 0.83 (0.72-0.96) | 8911/195296 | 0.85 (0.80-0.91) |
| P-int^§^ | 0.99 |  |  |  |

^*^ Denmark and Spain were excluded from this comparison (no data on marital status).

^†^ Includes women who reported that they were married, living together, divorced or widowed.

^‡^ Cox regression adjusted for menopausal status (premenopausal [Ref], perimenopausal/unknown menopause, postmenopausal and surgical postmenopausal [bilateral oophorectomy]), body mass index (<23, 23-24.9 [Ref], 25-29.9, 30-39.9, 40+ kg/m2), physical activity index (inactive [Ref], moderately inactive, moderately active, active, unknown), education status (none/primary school [Ref], technical/professional school, secondary school/longer education including university, or unknown), smoking status/intensity (never [Ref], current 1-15 cigarettes/day, current 16-25 cigarettes/day, current 26+ cigarettes/day, former quit ≤10 years, former quit 11-20 years, former quit >20 years, current pipe/cigar/occasional smoker, current/former missing timing, unknown), smoking duration (<10 [Ref], 10-<20, 20-<30, 30-<40, 40+ years, unknown), and stratified by age and study center.

^§^ P for interaction value was calculated using a likelihood ratio test to compare multivariate models with and without multiplicative interaction terms between parity and marital status.

**Table S4**. Association of number of FTPs with all-cause mortality in the EPIC study after stratifying by BMI

|  |  | **BMI <25 kg/m^2^** | | **BMI ≥25 kg/m^2^** | |
| --- | --- | --- | --- | --- | --- |
|  |  | **Cases/** | **Model HR^*^** | **Cases/** | **Model HR^*^** |
| **Number of FTPs**^†^ | **Value** | **non-cases** | **(95% CI)** | **non-cases** | **(95% CI)** |
|  | 1 | 1284/28829 | 1.00 (Ref) | 1023/17052 | 1.00 (Ref) |
|  | 2 | 2728/72757 | 0.91 (0.85-0.98) | 2298/49673 | 0.90 (0.83-0.97) |
|  | 3 | 1262/30669 | 0.87 (0.80-0.94) | 1267/25647 | 0.86 (0.79-0.94) |
|  | 4 | 461/7461 | 1.01 (0.91-1.13) | 586/8817 | 0.96 (0.87-1.07) |
|  | 5 | 135/1750 | 1.00 (0.83-1.20) | 215/2698 | 0.97 (0.84-1.14) |
|  | 6+ | 67/689 | 1.05 (0.81-1.36) | 170/1601 | 1.10 (0.93-1.31) |
|  | P-trend^‡^ |  | 0.53 |  | 0.56 |
|  | P-int^§^ | 0.93 |  |  |  |

Abbreviations: FTP, full term pregnancy.

^*^ Cox regression adjusted for menopausal status (premenopausal [Ref], perimenopausal/unknown menopause, postmenopausal and surgical postmenopausal [bilateral oophorectomy]), physical activity index (inactive [Ref], moderately inactive, moderately active, active, unknown), education status (none/primary school [Ref], technical/professional school, secondary school/longer education including university, or unknown), smoking status/intensity (never [Ref], current 1-15 cigarettes/day, current 16-25 cigarettes/day, current 26+ cigarettes/day, former quit ≤10 years, former quit 11-20 years, former quit >20 years, current pipe/cigar/occasional smoker, current/former missing timing, unknown), smoking duration (<10 [Ref], 10-<20, 20-<30, 30-<40, 40+ years, unknown), and stratified by age and study center.

^†^ Restricted to parous women. A FTP was defined as live births and stillbirths.

^‡^ P for trend values are based on continuous variables: the number of FTPs was modeled as 1-≥10.

^§^ P for interaction value was calculated using a likelihood ratio test to compare multivariate models with and without multiplicative interaction terms between categories of FTP numbers and BMI.

**Table S5**. Association of breastfeeding duration with all-cause mortality in the EPIC study after stratifying by the number of FTPs

|  |  | **≤2 FTPs** | | **>2 FTPs** | |
| --- | --- | --- | --- | --- | --- |
|  |  | **Cases/** | **Model HR**^†^ | **Cases/** | **Model HR**^†^ |
| **Breastfeeding, m^*^** | **Value** | **non-cases** | **(95% CI)** | **non-cases** | **(95% CI)** |
|  | <=1 | 733/16176 | 1.00 (Ref) | 216/3845 | 1.00 (Ref) |
|  | >1-<=3 | 1353/32028 | 1.02 (0.93-1.12) | 400/7907 | 0.95 (0.80-1.13) |
|  | >3-<=6 | 1347/31201 | 0.98 (0.89-1.07) | 499/10484 | 0.95 (0.80-1.12) |
|  | >6-<=12 | 1344/30815 | 0.94 (0.86-1.04) | 880/16987 | 0.97 (0.83-1.13) |
|  | >12-<=18 | 478/12874 | 0.88 (0.78-0.99) | 508/10445 | 0.89 (0.75-1.05) |
|  | >18 | 297/9083 | 0.90 (0.78-1.05) | 979/18866 | 1.00 (0.85-1.18) |
|  | P-trend^‡^ |  | 0.04 |  | 0.25 |
|  | P-int^§^ | 0.34 |  |  |  |

Abbreviations: FTP, full term pregnancy; m, months.

^*^ Among parous women who had ever breastfed (sum of all FTPs).

^†^ Cox regression adjusted for menopausal status (premenopausal [Ref], perimenopausal/unknown menopause, postmenopausal and surgical postmenopausal [bilateral oophorectomy]), body mass index (<23, 23-24.9 [Ref], 25-29.9, 30-39.9, 40+ kg/m2), physical activity index (inactive [Ref], moderately inactive, moderately active, active, unknown), education status (none/primary school [Ref], technical/professional school, secondary school/longer education including university, or unknown), smoking status/intensity (never [Ref], current 1-15 cigarettes/day, current 16-25 cigarettes/day, current 26+ cigarettes/day, former quit ≤10 years, former quit 11-20 years, former quit >20 years, current pipe/cigar/occasional smoker, current/former missing timing, unknown), smoking duration (<10 [Ref], 10-<20, 20-<30, 30-<40, 40+ years, unknown), and stratified by age and study center.

^‡^ P for trend values are based on continuous variables.

^§^ P for interaction value was calculated using a likelihood ratio test to compare multivariate models with and without multiplicative interaction terms between categories of breastfeeding and number of FTPs.
